# Chrysin enhances anticancer drug-induced toxicity mediated by the reduction of claudin-1 and 11 expression in a spheroid culture model of lung squamous cell carcinoma cells

**DOI:** 10.1038/s41598-019-50276-z

**Published:** 2019-09-24

**Authors:** Ryohei Maruhashi, Hiroaki Eguchi, Risa Akizuki, Shohei Hamada, Takumi Furuta, Toshiyuki Matsunaga, Satoshi Endo, Kenji Ichihara, Akira Ikari

**Affiliations:** 1 Laboratory of Biochemistry, Department of Biopharmaceutical Sciences, Gifu Pharmaceutical University, Gifu, 501-1196 Japan; 2 Department of Pharmaceutical Chemistry, Kyoto Pharmaceutical University, Yamashina-ku, Kyoto 607-8414 Japan; 3 Education Center of Green Pharmaceutical Sciences, Gifu Pharmaceutical University, Gifu, 502-8585 Japan; 4 Nagaragawa Research Center, API Co., Ltd., Gifu, 502-0071 Japan

**Keywords:** Non-small-cell lung cancer, Tight junctions

## Abstract

The aberrant expression of claudins (CLDNs), which are tight junctional proteins, is seen in various solid tumors, but the regulatory mechanisms and their pathophysiological role are not well understood. Both CLDN1 and CLDN11 were highly expressed in human lung squamous cell carcinoma (SCC). Chrysin, found in high concentration in honey and propolis, decreased CLDN1 and CLDN11 expression in RERF-LC-AI cells derived from human lung SCC. The phosphorylation level of Akt was decreased by chrysin, but those of ERK1/2 and c-Jun were not. LY-294002, an inhibitor of phosphatidylinositol 3-kinase, inhibited the phosphorylation of Akt and decreased the expression levels of CLDN1 and CLDN11. The association between phosphoinositide-dependent kinase 1 (PDK1) and Akt was inhibited by chrysin, but the phosphorylation of PDK1 was not. Immunoprecipitation and quartz-crystal microbalance assays revealed that biotinylated-chrysin binds directly to Akt. The knockdown of CLDN1 and CLDN11 using small interfering RNAs increased the transepithelial flux of doxorubicin (DXR), an anthracycline anticancer drug. Similarly, both chrysin and LY-294002 increased DXR flux. Neither CLDN1 knockdown, CLDN11 knockdown, nor chrysin changed the anticancer drug-induced cytotoxicity in a two-dimensional culture model, whereas they enhanced cytotoxicity in a spheroid culture model. Taken together, chrysin may bind to Akt and inhibit its phosphorylation, resulting in the elevation of anticancer drug-induced toxicity mediated by reductions in CLDN1 and CLDN11 expression in RERF-LC-AI cells. We suggest that chrysin may be useful as an adjuvant chemotherapy in lung SCC.

## Introduction

Lung cancer is the leading cause of cancer death worldwide, and non-small-cell lung cancer (NSCLC) accounts for over 80% of lung cancers. NSCLC generally develops without symptoms or remarkable physical findings, and often presents at stages too late for surgical intervention. Approximately half of patients are diagnosed at a late stage. In addition, advanced NSCLC commonly exhibits resistance to chemotherapy and radiation^1^. Therefore, conventional systemic chemotherapy shows lower response rates in patients with NSCLC. Squamous cell carcinoma (SCC) is one histological subtype of NSCLC, accounting for 25–30% of NSCLC cases^2^. Recently, immune checkpoint inhibitors (ICIs) have been shown to improve overall survival in advanced NSCLC compared with standard treatment^3^. However, the efficacy of ICIs as a monotherapy for NSCLC is seen in only approximately 20% of patients^4^.

Cisplatin (CDDP), carboplatin, and oxaliplatin, are platinum-based drugs that are widely used in lung cancer chemotherapy. However, long-term application of chemotherapy may result in the development if acquired resistance in cells that were sensitive originally. Furthermore, acquired drug resistance can confer cross-resistance to a wide range of compounds that have no obvious structural or functional similarities, thereby causing inefficient treatment. Over 50% of patients undergoing lung cancer surgery acquire a chemoresistant phenotype^5^. A variety of mechanisms including mutation in the target molecule of anticancer drugs, induction of drug efflux pumps and drug-metabolizing enzymes, and DNA epigenetic states are involved in the development of drug resistance^6^. The establishment of a tumor microenvironment, which consists of leukocytes, extracellular matrix, endothelial cells, and so on, is also involved in the development of chemoresistance^7^, but the molecular mechanisms remain elusive. A spheroid is a three-dimensional (3D) *in vitro* tumor model that resembles the *in vivo* situation^8^. We recently reported that claudin-1 (CLDN1), CLDN2, and occludin, components of tight junctions (TJs), decrease chemosensitivity to doxorubicin (DXR), an anthracycline anticancer drug, in 3D-cultured lung adenocarcinoma A549 cells^9,10^. The expression levels of CLDN3, 4, 5, 7, and 18 are down-regulated in human lung SCC tissue and in RERF-LC-AI cells, which are derived from human lung SCC, compared with normal lung tissue, whereas CLDN1 is highly expressed. However, the pathophysiological role of the abnormal expression of CLDNs is not yet fully understood.

Flavonoids are dietary phenolic compounds found ubiquitously in plant foods such as fruits and vegetables (26). Most flavonoids have anti-oxidant, anti-proliferative, and anti-tumor activities^11^. Chrysin is a natural flavonoid contained in various plants and propolis. Chrysin inhibits proliferation and induces apoptosis by inhibiting Akt activation in NSCLC cells^12,13^. The chemopreventive effects of chrysin have been reported in hepatocellular carcinoma^14^, anaplastic thyroid cancer^15^, breast carcinoma^16^, and prostate carcinoma xenograft mice models^17^. In addition, chrysin has the potential to enhance and improve the sensitivity of NSCLC cells to anticancer drugs^18^. However, the anticancer mechanisms of chrysin have not been fully elucidated.

Human SCC tissue and RERF-LC-AI cells derived from human lung SCC express not only CLDN1, but also CLDN11 at high levels. Therefore, we investigated their pathophysiological roles and searched for compounds that can decrease CLDN1 and CLDN11 expression. Chrysin decreased the expression of CLDN1 and CLDN11 mediated by the inhibition of Akt. The direct interaction of chrysin with Akt was observed by the immunoprecipitation and quartz crystal microbalance (QCM) assays. Chrysin enhanced anticancer agent-induced toxicity in a 3D spheroid culture model with RERF-LC-AI cells. Our data indicate that chrysin is a potential compound for the adjuvant treatment of human SCC.

## Results

### Expression of CLDN1 and CLDN11 in human lung SCC and RERF-LC-AI cells

**Figure 1:**
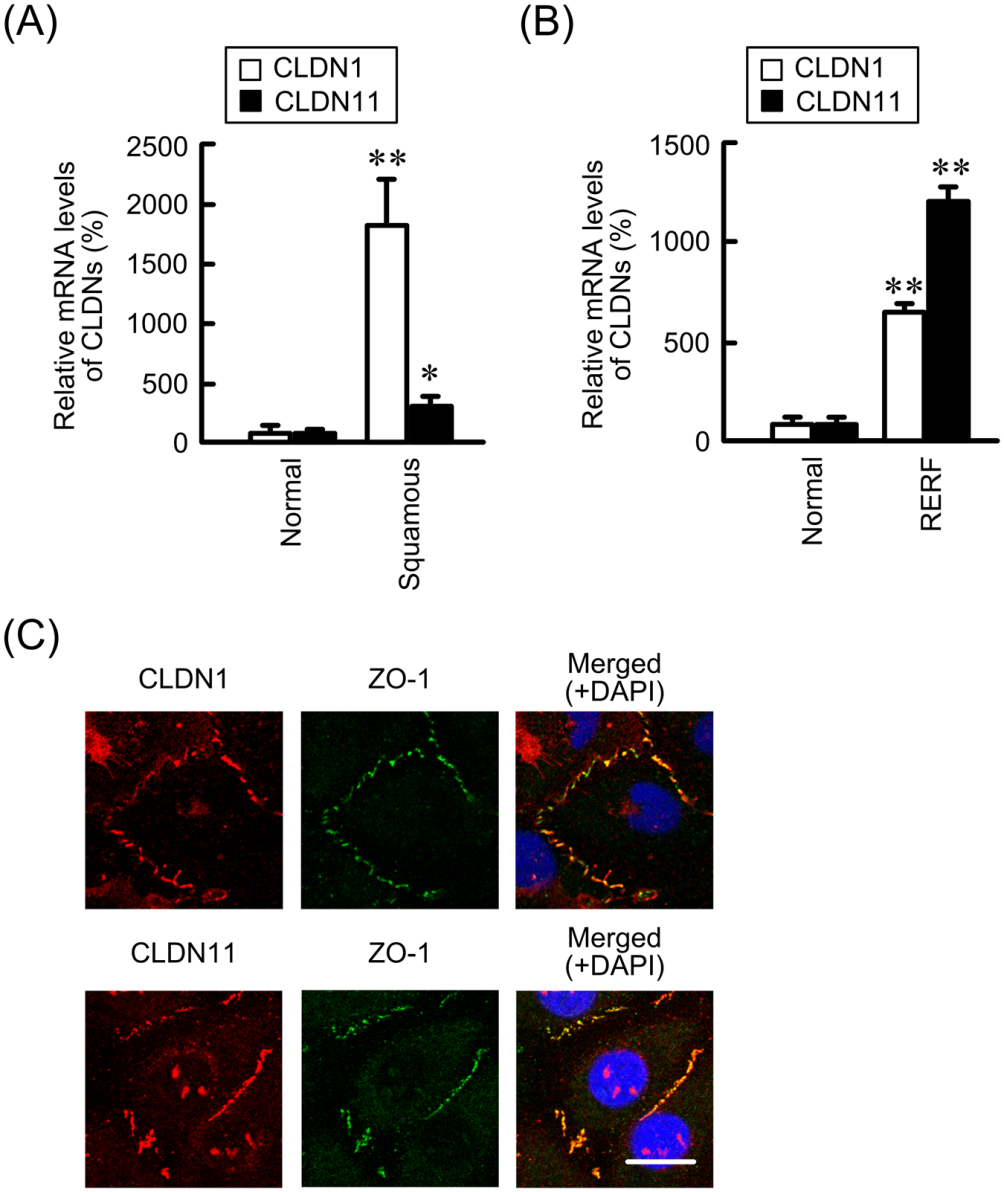
Expression of CLDN1 and CLDN11 in human normal lung and SCC cells. (**A**) The expression levels of CLDN1 and CLDN11 mRNAs in human lung SCC tissue are shown as a percentage of the values in normal lung tissues. (**B**) The expression levels of CLDN1 and CLDN11 mRNAs in RERF-LC-AI cells, derived from human lung SCC are shown as a percentage of the values in normal lung tissues. (**C**) Immunofluorescence staining with anti-CLDN1, anti-CLDN11 (red), and anti-ZO-1 (green) antibodies was performed. The right-hand images show merged pictures with DAPI (blue). Scale bar represents 10 µm. n = 3–4. **P < 0.01 and *P < 0.05 compared with normal. NS, *P* > 0.05.

We reported previously that CLDN1 is highly expressed in human lung SCC tissue and RERF-LC-AI cells, whereas the expression levels of CLDN3, CLDN4, CLDN5, CLDN7, and CLDN18 were lower than those in normal tissue^19^. Here, we found that CLDN11 is also highly expressed in human lung SCC tissue and RERF-LC-AI cells (Fig. 1). CLDNs are scaffolded by zonula occludens-1 (ZO-1), which interacts with the actin cytoskeleton^20,21^. Both CLDN1 and CLDN11 were colocalized with ZO-1, but the images showed punctate staining of CLDNs and ZO-1 in the cell-cell border area.

### Effects of knockdown of CLDN1 and CLDN11 on sensitivity to anticancer drugs

**Figure 2:**
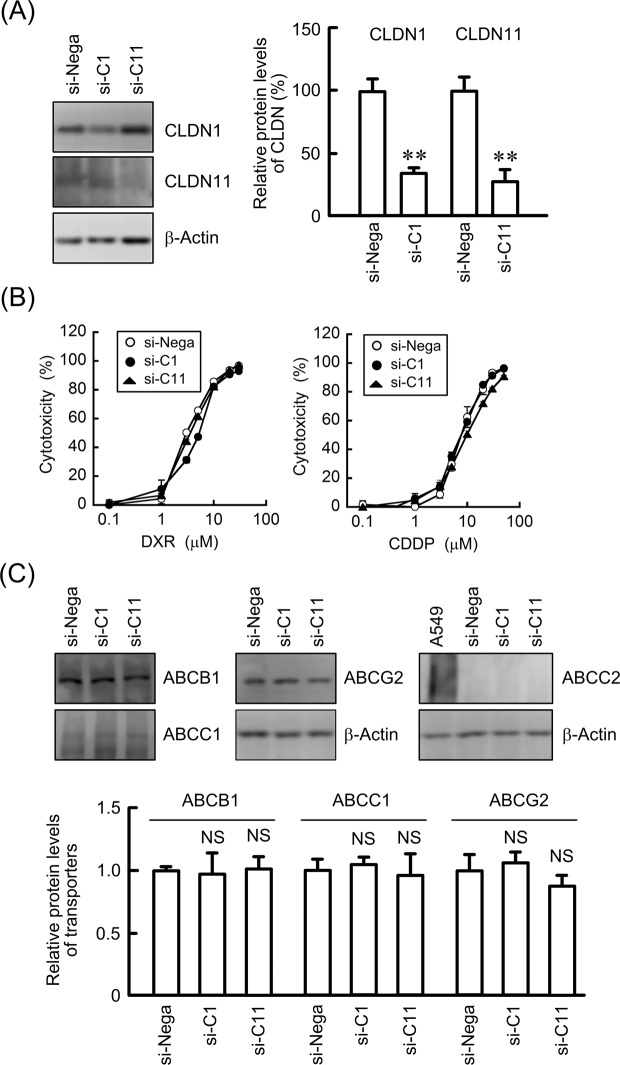
Effect of knockdown of CLDN1 and CLDN11 on the cytotoxicity of anticancer drugs. RERF-LC-AI cells were transfected with siRNAs for a negative control (si-Nega), CLDN1 (si-C1), or CLDN11 (si-C11). (**A**) The expression levels of CLDN1, CLDN11, and β-actin in cell lysates were examined by western blotting. The protein levels are shown as a percentage of values in the negative control siRNA (si-Nega). (**B**) Cytotoxicity was measured using WST-1 assays. (**C**) The expression levels of ABCB1, ABCC1, ABCG2, ABCC2, and β-actin in cell lysates were examined by western blotting. A549 cells were used as a positive control in the detection of ABCC2. The protein levels without ABCC2 are shown as a percentage of values in the negative control siRNA (si-Nega). The full-length blot images are shown in Supplementary Fig. S1. n = 3–6. **P < 0.01 and NS, *P* > 0.05 compared with the negative control siRNA.

The pathophysiological roles of CLDN1 and CLDN11 have not been fully clarified in lung SCC cells. We examined the effects of CLDN1 and CLDN11 knockdown on anticancer agent-induced cytotoxicity. The introduction of small interfering RNAs (siRNAs) for CLDN1 and CLDN11 suppressed the protein expression of CLDN1 and CLDN11, respectively (Fig. 2A). DXR and CDDP increased cytotoxicity in a dose-dependent manner (Fig. 2B). Neither CLDN1 nor CLDN11 knockdown significantly changed the sensitivity to DXR and CDDP. Anticancer-induced cytotoxicity was diminished by the activation of their extrusion through ABC transporters^22^. Neither CLDN1 nor CLDN11 knockdown significantly changed the protein levels of ABCB1, ABCC1, or ABCG2 (Fig. 2C). The expression of ABCC2 was detected in positive control A549 cells, but not in RERF-LC-AI cells. These results indicated that neither CLDN1 nor CLDN11 is involved in chemosensitivity in this 2D culture model.

### Effects of sodium caprate (CA) and knockdown of CLDN on transepithelial permeability

**Figure 3:**
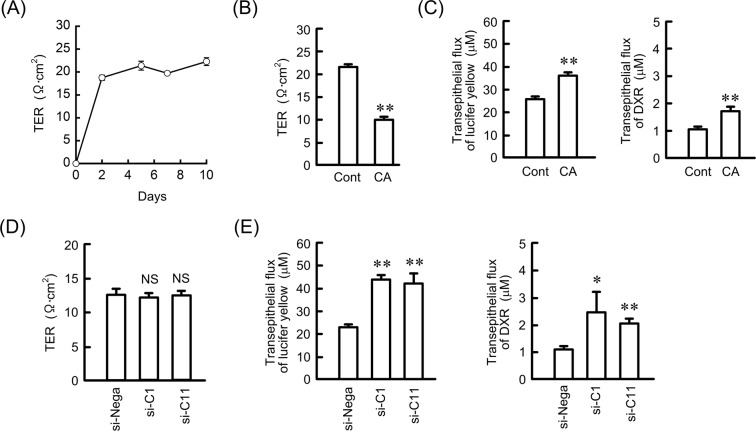
Effects of CA and knockdown of CLDN on tight junction permeability. RERF-LC-AI cells were plated on Transwell inserts. (**A**) TER was analyzed on days 0, 2, 5, 7, and 10 using a volt ohmmeter. (**B**,**C**) After culturing for 4 days, the cells were incubated in the presence or absence of 10 mM CA for 2 h. TER was analyzed using a volt ohmmeter. Transepithelial lucifer yellow and DXR fluxes were analyzed using a fluorescence spectrometry. (**D**,**E**) The cells were transfected with siRNAs for negative control, CLDN1, or CLDN11. TER and transepithelial flux were analyzed. n = 3–4. **P < 0.01, *P < 0.05, and NS, *P* > 0.05 compared with the negative control siRNA.

CLDN subtypes can form homo- or heterophilic interactions between adjacent cells and regulate paracellular solute and ion transport^23–25^. Transepithelial electrical resistance (TER) was increased after days 2 and made a plateau phase (Fig. 3A). TER was significantly decreased by CA, a TJs modulator (Fig. 3B). In addition, transepithelial fluxes of lucifer yellow, a fluorescent marker for the paracellular pathway, and DXR were increased by CA (Fig. 3C). Both TER and transepithelial fluxes of lucifer yellow were changed by CA, but RERF-LC-AI cells did not form continuous TJs and their TJ barrier was incomplete. Both CLDN1 and CLDN11 knockdown increased transepithelial permeability to DXR without changing TER (Fig. 3D,E). These results indicated that CLDN1 and CLDN11 may not be involved in the regulation of paracellular ion permeability, but they may suppress paracellular permeability for small molecules.

### Increase in DXR sensitivity by knockdown of CLDN1 and CLDN11 in a 3D spheroid model

**Figure 4:**
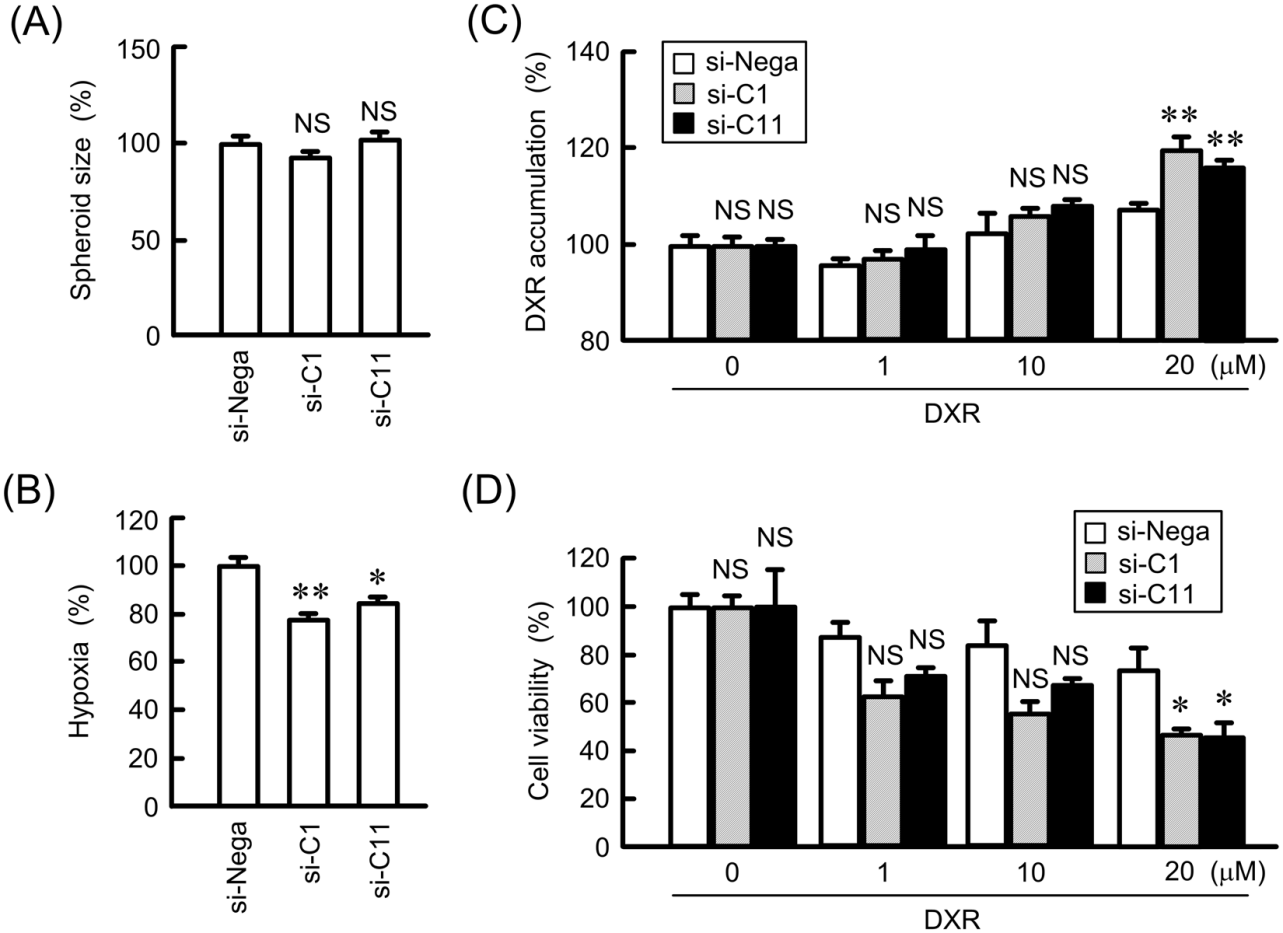
Effect of knockdown of CLDN1 and CLDN11 on hypoxia levels and anticancer drug-induced toxicity in spheroids. RERF-LC-AI cells were plated on PrimeSurface96V multi-well plates and transfected with siRNAs for negative control, CLDN1, or CLDN11. (**A**,**B**) After treating the cells with 2 μM LOX-1 for 24 h, fluorescence images were acquired. The spheroid size and fluorescence intensities of LOX-1 are represented as a percentage of values in the negative control siRNA. (**C**) The cells were incubated with DXR for 60 min. The fluorescence intensities of DXR in spheroids are shown as a percentage of negative siRNA. (**D**) After treating the cells with DXR for 24 h, the viability of spheroid cells were measured. These values are represented as a percentage of a values in the negative control siRNA. n = 4–6. **P < 0.01, *P < 0.05, and NS, *P* > 0.05 compared with negative control siRNA.

Neither CLDN1 nor CLDN11 changed spheroid sizes, but they significantly decreased hypoxia levels (Fig. 4A,B). The fluorescence intensity of DXR in the spheroids was increased in a dose dependent manner, indicating that DXR accumulated in the spheroids (Fig. 4C). The accumulation of DXR was significantly enhanced by CLDN1 and CLDN11 knockdown. DXR decreased the viability of spheroid cells in a dose-dependent manner, which was enhanced by CLDN1 and CLDN11 knockdown (Fig. 4D). These results indicated that both CLDN1 and CLDN11 may be involved in chemoresistance in 3D spheroid cells.

### Effect of luteolin and chrysin on the expression of CLDN1 and CLDN11 in RERF-LC-AI cells

**Figure 5:**
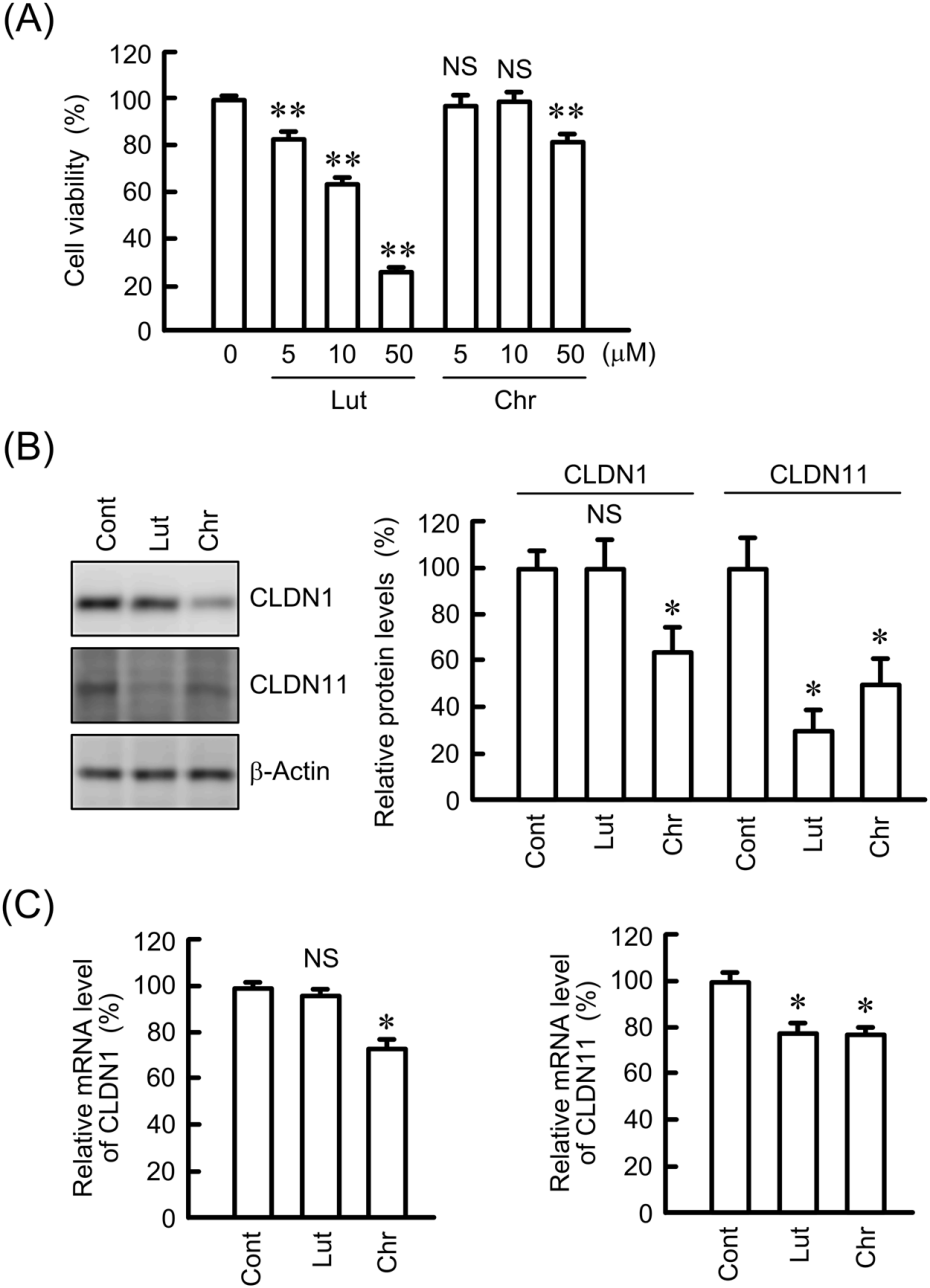
Effect of luteolin and chrysin on the expression levels of CLDN1 and CLDN11. (**A**) RERF-LC-AI cells were treated with luteolin or chrysin at the indicated concentrations for 24 h. Cytotoxicity was measured using WST-1 assays. (**B**) The cells were treated with 10 μM luteolin (Lut) or 10 μM chrysin (Chr) for 24 h. The expression levels of CLDN1, CLDN11, and β-actin in cell lysates were examined by western blotting. The protein levels of CLDN1 and CLDN11 are shown as a percentage of values in the control cells (Cont). The full-length blot images are shown in Supplementary Fig. S2. (**C**) The cells were treated with 10 μM luteolin or 10 μM chrysin for 6 h. The mRNA levels of CLDN1 and CLDN11 are shown as a percentage of the values in control cells. n = 3–4. **P < 0.01, *P < 0.05, and NS, *P* > 0.05 compared with 0 μM or the control.

Many flavonoids have been reported to have anticancer activity^26^. We examined the effect of some flavonoids contained in extracts of propolis on CLDN expression. Cell viability was decreased by luteolin in a dose-dependent manner between 5 and 50 μM and by chrysin above 50 μM (Fig. 5A). The protein and mRNA levels of CLDN11 were decreased by both 10 μM luteolin and 10 μM chrysin, whereas those of CLDN1 were decreased by chrysin only (Fig. 5B,C). The protein levels of CLDN1 were not decreased by quercetin or kaempferol (data not shown). Therefore, we tried to clarify the molecular mechanism of action of chrysin.

### Involvement of Akt in the regulation of CLDN1 expression

**Figure 6:**
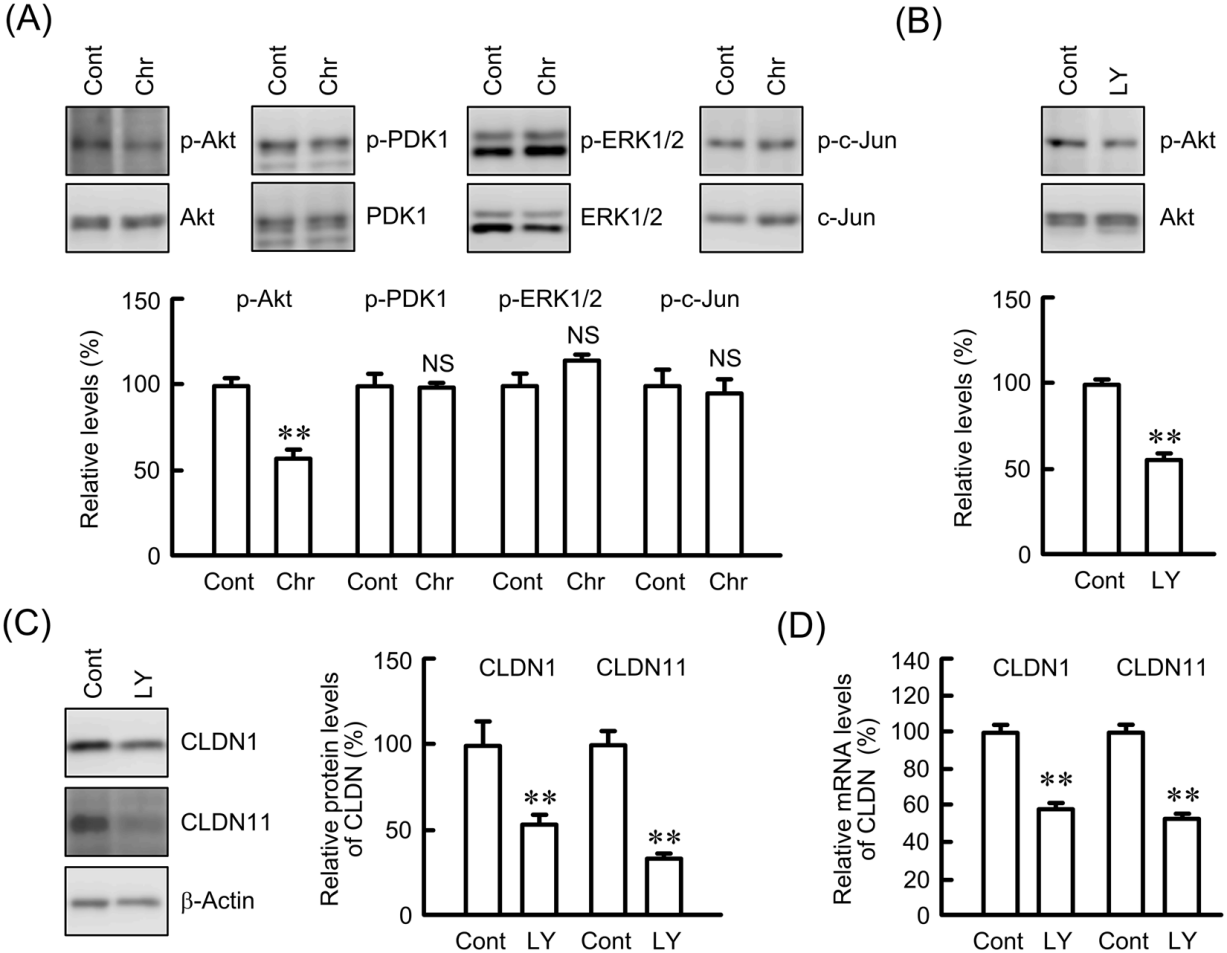
Effect of LY-294002 on the expression of p-Akt and CLDNs. (**A**) The expression levels of p-Akt, Akt, p-PDK1, PDK1, p-ERL1/2, ERK1/2, p-c-Jun, and c-Jun in cell lysates were examined by western blotting. The levels of p-Akt, p-PDK1, p-ERK1/2, and p-c-Jun are shown as a percentage of the values in non-treated cells (Cont). (**B**) RERF-LC-AI cells were treated with or without 20 μM LY-294002 (LY) for 1 h. The expression levels of p-Akt are shown as a percentage of values in the absence of LY-294002. (**C**) The cells were treated with or without 20 μM LY-294002 for 24 h. The expression levels of CLDN1, CLDN11, and β-actin in cell lysates were examined by western blotting. The protein levels of CLDN1 and CLDN11 are shown as a percentage of values in the absence of LY-294002. The full-length blot images are shown in Supplementary Fig. S3. (**D**) The cells were treated with and without 20 μM LY-294002 for 6 h. The mRNA levels of CLDN1 and CLDN11 are shown as a percentage of values in the control (Cont). n = 3–4. **P < 0.01 and NS, *P* > 0.05 compared with the control cells.

There are no reports on what intracellular signaling factors are involved in CLDN1 and CLDN11 expression in lung SCC cells. Chrysin did not change p-ERK1/2 or p-c-Jun levels, whereas it decreased p-Akt levels (Fig. 6A). The phosphorylation of Akt is upregulated by phosphoinositide-dependent kinase 1 (PDK1), a kinase functioning downstream of phosphatidylinositol 3-kinase (PI3K). However, p-PDK1 levels were not changed by chrysin. The total amounts of Akt, PDK1, ERK1/2, and c-Jun were not changed by chrysin. LY-294002, an inhibitor of the PI3K/Akt signaling pathway, significantly decreased p-Akt, CLDN1, and CLDN11 levels (Fig. 6B,C). In addition, the mRNA levels of CLDN1 and CLDN11 were decreased by LY-294002 (Fig. 6D). These results coincided with those using chrysin.

### Inhibition of the association between PDK1 and Akt by chrysin

**Figure 7:**
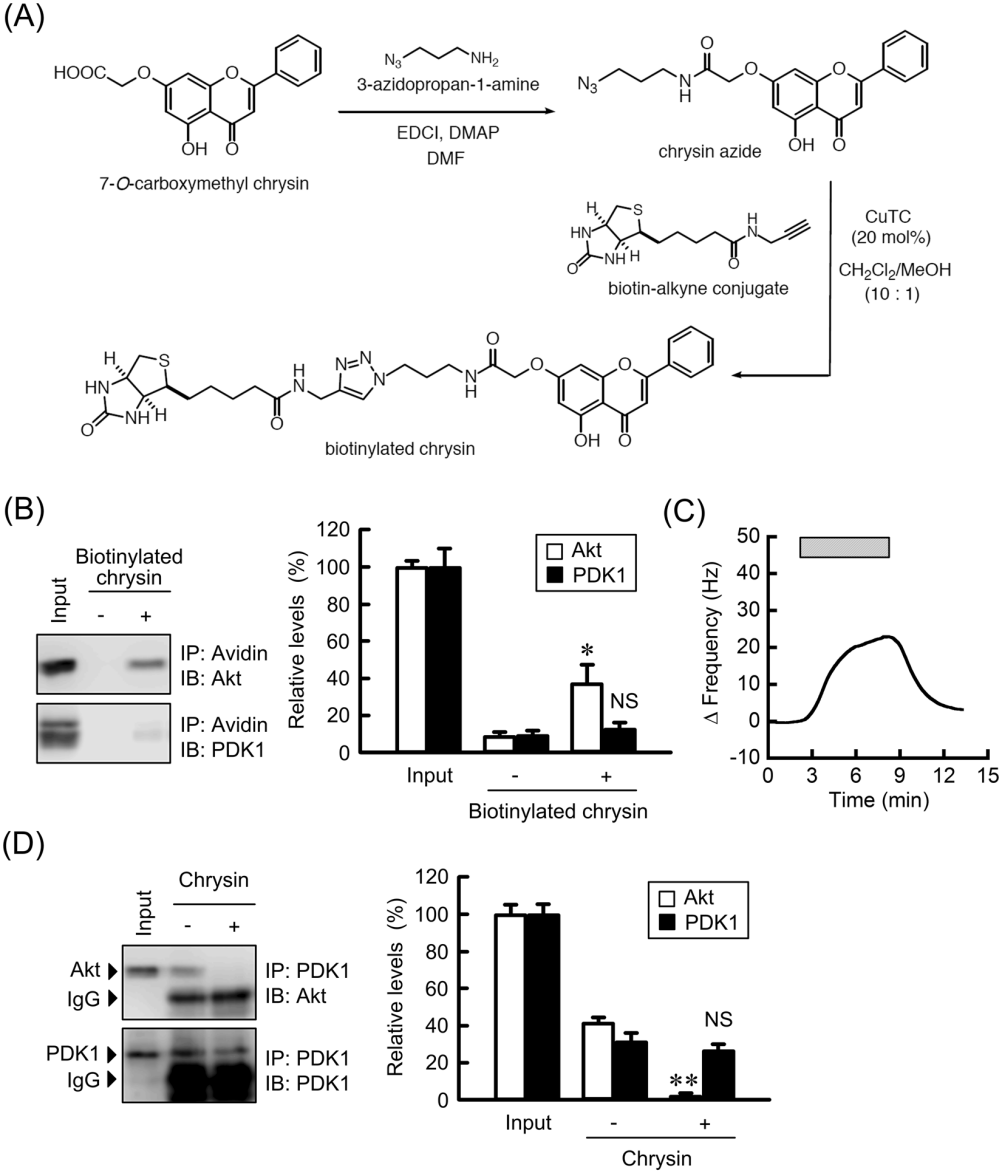
Association of chrysin with Akt. (**A**) Reaction scheme for the synthesis of biotinylated chrysin. (**B**) Cell lysates were incubated with or without biotinylated-chrysin in the presence of avidin agarose. Precipitated proteins were subjected to western blotting. The protein levels of precipitated Akt and PDK1 are shown as a percentage of values in the input. The full-length blot images are shown in Supplementary Fig. S4. (**C**) The QCM frequency was measured every 1 s. Then, 10 ng/mL biotinylated-chrysin was applied at the time period indicated by the hatched box. (**D**) Cell lysates prepared from the cells treated with or without 10 μM chrysin were incubated with protein G sepharose beads and anti-PDK1 antibody. Precipitated proteins were subjected to western blotting. IgG indicates the band of heavy chain of immunoglobulin G. The protein levels of precipitated Akt and PDK1 are shown as a percentage of values in the input. The full-length blot images are shown in Supplementary Fig. S4. n = 3–4. **P < 0.01 and NS, *P* > 0.05 compared without biotinylated-chrysin or chrysin.

To clarify the direct interaction of chrysin, we synthesized biotinylated-chrysin (Fig. 7A). In immunoprecipitation assays, biotinylated-chrysin bound to Akt, but not to PDK1 (Fig. 7B). In addition, QCM analysis showed that biotinylated-chrysin bound to human recombinant Akt (Fig. 7C). Akt was associated with PDK1, which was significantly inhibited by chrysin (Fig. 7D). In contrast, the level of immunoprecipitated PDK1 was not changed by chrysin. These results indicated that chrysin may inhibit phosphorylation of Akt and interaction of Akt with PDK1 mediated by direct association with Akt.

### Effects of chrysin on sensitivity and transepithelial permeability to anticancer drugs

**Figure 8:**
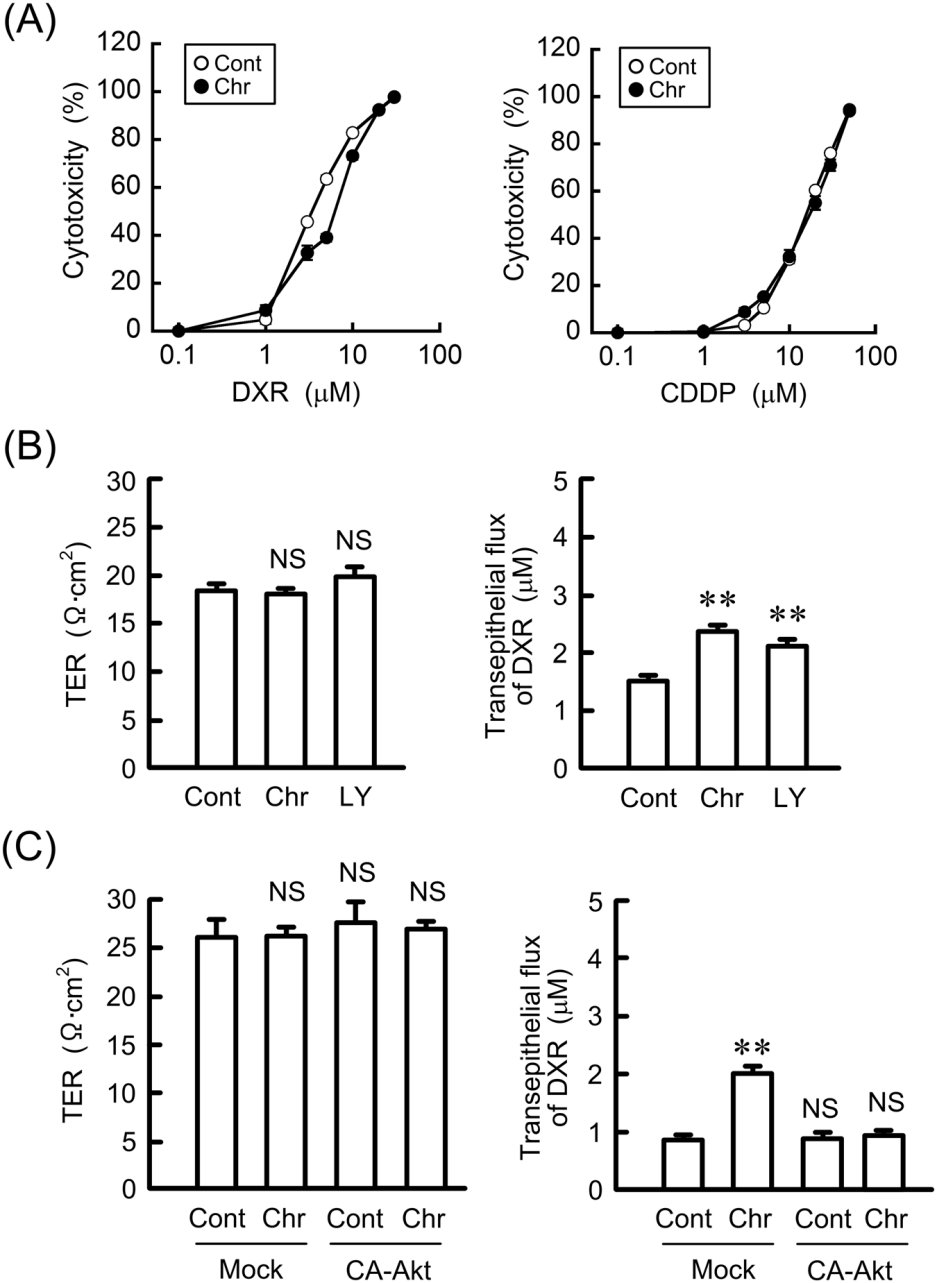
Effects of chrysin and CA-Akt on anticancer drug-induced cytotoxicity and tight junction permeability. (**A**) RERF-LC-AI cells were treated with DXR or CDDP in the presence or absence of 10 μM chrysin (Chr) for 24 h. Cytotoxicity was measured using WST-1 assays. (**B**) The cells were plated on transwell inserts and treated with 10 μM chrysin or 20 μM LY-294002. TER and transepithelial DXR flux were analyzed using a volt ohmmeter and fluorescence spectrometry, respectively. (**C**) The cells were transfected with mock or CA-Akt vector, and then treated with 10 μM chrysin. TER and transepithelial DXR flux were analyzed. n = 3–4. **P < 0.01, *P < 0.05, and NS, *P* > 0.05 compared with the control.

As shown in Fig. 5A, chrysin did not show cytotoxicity at 10 μM. Chrysin slightly suppressed the DXR-induced cytotoxicity at 3–10 μM, whereas it did not affect CDDP-induced cytotoxicity (Fig. 8A). Next, we examined the effects of chrysin and LY-294002 on transepithelial permeability. Both chrysin and LY-294002 increased transepithelial permeability to DXR without changing TER (Fig. 8B). These results coincided with those using CLDN1 and CLDN11 knockdown.

### Increase in DXR and CDDP toxicities of spheroid cells by chrysin

**Figure 9:**
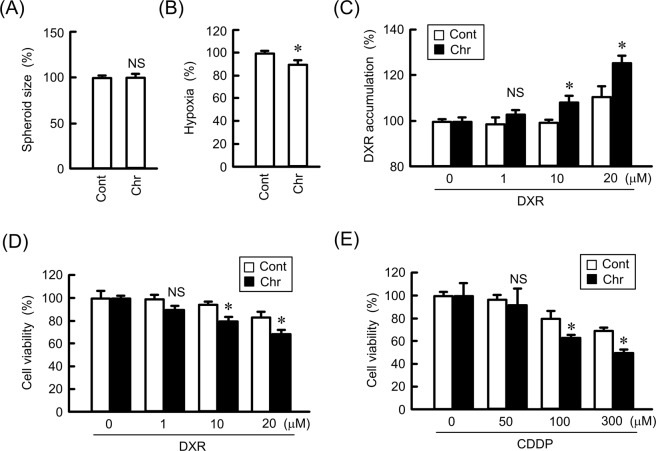
Increase in anticancer-induced toxicity by chrysin in a spheroid model. RERF-LC-AI cells were plated on PrimeSurface96V multi-well plates, and then treated with or without 10 μM chrysin (Chr) for 24 h. (**A**,**B**) The spheroid size and fluorescence intensity of LOX-1 are represented as a percentage of the values in the control (Cont). (**C**) The cells were incubated with DXR for 60 min at the concentrations indicated. The fluorescence intensities of DXR in spheroids are shown as a percentage of the values in 0 μM. (**D**,**E**) After treating the cells with DXR or CDDP for 24 h at the concentrations indicated, the viability of spheroid cells was measured. These values are represented as a percentage of the control. n = 3–4. *P < 0.05 and NS, *P* > 0.05 compared with the control.

**Figure 10:**
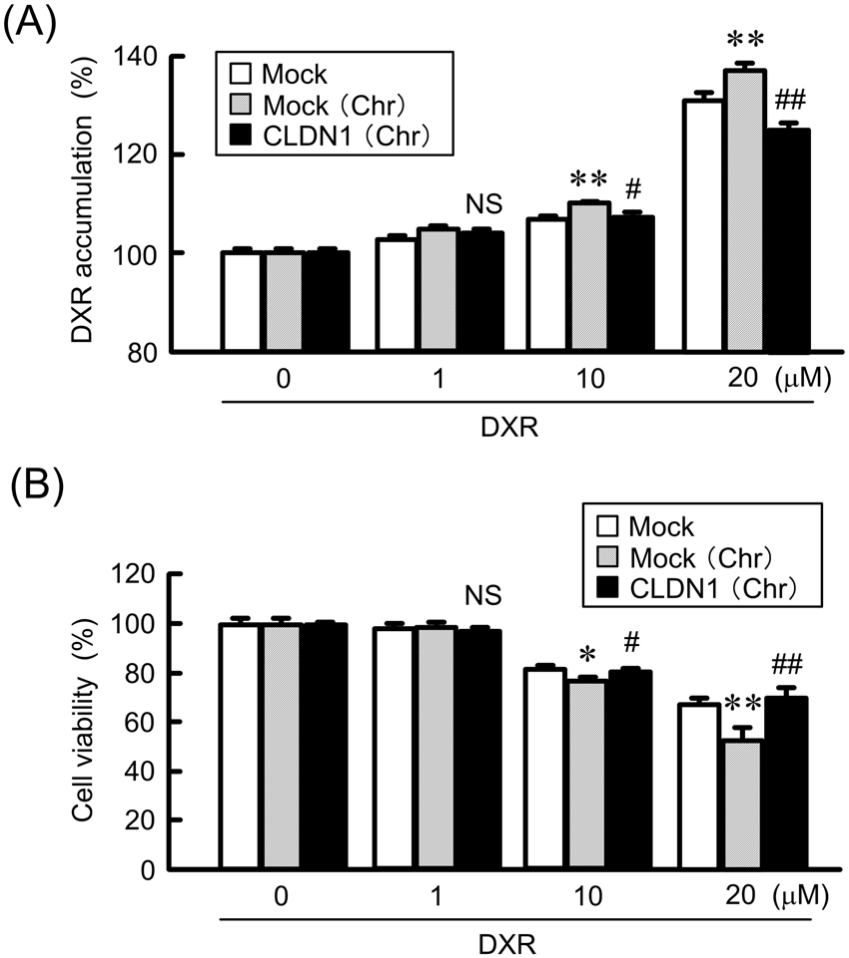
Inhibition of chrysin-induced elevation of toxicity by CLDN1 overexpression in a spheroid model. RERF-LC-AI cells were plated on PrimeSurface96V multi-well plates, and then transfected with mock or CLDN1 expression vector. (**A**) The cells were incubated with DXR for 60 min at the concentrations indicated in the presence or absence of 10 μM chrysin. The fluorescence intensities of DXR in spheroids are shown as a percentage of the values in 0 μM. (**B**) After treating the cells with DXR for 24 h at the concentrations indicated in the presence or absence of chrysin, the viability of spheroid cells was measured. These values are represented as a percentage of mock. n = 3–4. **P < 0.01 and *P < 0.05 compared with mock. ^##^*P* < 0.01, ^#^*P* < 0.05 and NS, *P* > 0.05 compared with mock (Chr).

Chrysin did not change spheroid size, but it significantly decreased hypoxia levels (Fig. 9A,B). The accumulation of DXR in the spheroids was significantly enhanced by chrysin (Fig. 9C). DXR and CDDP decreased the viability of spheroid cells in a dose-dependent manner, which was enhanced by chrysin (Fig. 9D,E). These results coincided with those using CLDN1 and CLDN11 knockdown. Chrysin may enhance the sensitivity to anticancer drugs in 3D spheroid cells mediated by the reduction in CLDN1 and CLDN11 expression. To support the idea, we examined the effect of CLDN1 overexpression on the accumulation and toxicity of DXR. The chrysin-induced elevation of accumulation and toxicity of DXR was significantly inhibited by CLDN1 overexpression (Fig. 10).

## Discussion

Normal lung epithelia expresses CLDN1, 3, 4, 5, 7, and 18, but not CLDN11^27,28^. We reported previously that the expression of CLDN1 is upregulated in human SCC tissue and RERF-LC-AI cells compared with normal tissue, whereas CLDN3, 4, 5, 7, and 18 are downregulated^19^. Here, we found that CLDN11 is highly expressed in SCC (Fig. 1). Immunofluorescence and TER measurements showed the cells form incomplete TJs. However, transepithelial fluxes of lucifer yellow and DXR were increased by the treatment with CA or introduction of siRNA for CLDN1 and CLDN11, suggesting that the cells can form partially functional TJs barrier. The elevation of CLDN1 expression was reported in various carcinoma tissues including colon^29^, stomach^30^, and pancreas^31^. Similar to our results, Paschoud *et al*.^32^ reported that the mRNA and protein levels of CLDN1 in SCC are higher than those in parenchyma. However, their report indicated no correlation between the expression of CLDN1 and the clinicopathological characteristics of SCC.

The downregulation of CLDN3 and CLDN7 is associated with poor prognosis and poor survival in patients with SCC, respectively^33,34^. The overexpression of CLDN5, 7, and 18 suppresses cell cycle progression from G1 to S phase in RERF-LC-AI cells, indicating that these CLDNs are involved in the regulation of SCC cell proliferation^19^. In contrast, the pathophysiological roles of CLDN1 and CLDN11 in SCC are not fully understood. We reported recently that the expression of CLDN1 is exaggerated by CDDP resistance in A549 cells^10^. The knockdown of CLDN1 and CLDN11 expression by siRNA increased the transepithelial flux of DXR, but it did not significantly change anticancer drug-induced toxicity or the expression of ABC transporters in a 2D culture model of RERF-LC-AI cells (Figs 2 and 3), suggesting that neither CLDN1 nor CLDN11 are directly involved in chemoresistance against anticancer drugs.

The PI3K/Akt signaling pathway is mainly involved in the regulation of cell survival and apoptosis^35^, and the activation of Akt has been reported in over 60% of NSCLC patients^36^. Both CLDN1 and CLDN11 expression levels were decreased by LY-294002 (Fig. 6), suggesting that their expression is upregulated by Akt in SCC cells. Similarly, the expression of CLDN1 is upregulated by Akt in A549^19^ and cervical adenocarcinoma cells^37^. In contrast, an inverse relationship between phosphorylation of Akt and CLDN1 expression is reported in esophageal SCC cells^38^. The regulatory mechanism of CLDN1 expression may differ according to the type of carcinoma. On the other hand, there are no reports showing whether Akt is involved in the regulation of CLDN11 expression. Notably, chrysin decreased both CLDN1 and CLDN11 expression (Fig. 5). Furthermore, chrysin significantly decreased p-Akt levels, but did not change p-ERK1/2 and p-c-Jun levels. We suggest that chrysin decreases both CLDN1 and CLDN11 in RERF-LC-AI cells mediated by the inhibition of p-Akt.

Chrysin shows a preventive effect on cancer mediated by various mechanisms including reduction in the activities of cytochrome P450-dependent monooxygenases, induction of the activity of antioxidant and detoxification enzymes, inhibition of cellular proliferation, and induction of apoptosis^39^. The phenotype of cancer cells is regulated by diverse signaling molecules including the Akt pathway. Chrysin decreased p-Akt levels without affecting the total amount of Akt or phosphorylation of PDK1 (Fig. 6A). The inhibition of Akt by chrysin has been reported in A549^12^, breast cancer^40^ and leukemia cells^41^. Therefore, it is suggested that chrysin binds to Akt, although there was no direct evidence showing this as yet. Immunoprecipitation and QCM assays revealed that biotinylated chrysin can bind directly to Akt (Fig. 7B,C). The association between PDK1 and Akt was inhibited by chrysin (Fig. 7D). Thus, our data provide the first indication that chrysin can interact directly with Akt, resulting in inhibition of the phosphorylation of Akt and association of PDK1 with Akt.

The knockdown of CLDN1 and CLDN11 by siRNA enhances DXR accumulation and DXR-induced toxicity in 3D spheroid cells (Fig. 4). Similar results were observed using treatment with chrysin (Fig. 9B). Transepithelial flux of DXR was negatively regulated by CLDN1 and CLDN11. The accumulation and toxicity of DXR was significantly suppressed by CLDN1 overexpression (Fig. 10). We suggest that CLDN1 or CLDN11 overexpression impaired tumor sensitivity to anticancer drugs mediated by interference with penetration of the drugs into inner areas of the spheroid. Another explanation is that both CLDN1 and CLDN11 induce chemoresistant characteristics of cancer cells. Hypoxia levels of spheroid were inversely changed by CLDN1 and CLDN11 expression (Fig. 4). Cancer cells form microenvironment *in vivo*, and the inner cells of the microenvironment are exposed to nonlethal hypoxia and oxidative stress, which has significant effects on tumor progression and treatment efficacy. These findings raise a possibility that the alleviation of hypoxic conditions by decreasing in the expression of CLDN1 and CLDN11 improves chemosensitivity in spheroid cells. High interstitial fluid pressure, increased collagen production, and poorly formed vasculature exacerbate hypoxia^42^. However, it is unknown what mechanisms are involved in the establishment of hypoxic conditions *in vitro* in spheroids. Further studies are needed to clarify how CLDN and chrysin improve them, but chrysin may be useful to improve hypoxia and suppress the malignancy of SCC cells.

In conclusion, we found that human SCC tissue and RERF-LC-AI cells exhibit high expression levels of not only CLDN1 but also CLDN11 compared with normal tissue. Chrysin inhibited the phosphorylation of Akt and decreased the expression levels of CLDN1 and CLDN11 similar to LY-294002. Immunoprecipitation and QCM assays showed that chrysin binds directly to Akt and inhibits the association of PDK1 with Akt. Chrysin increased transepithelial flux of DXR without affecting TER. In addition, chrysin did not change anticancer agent-induced toxicity in a 2D model, but it enhanced toxicity in a 3D spheroid model. Our data indicate that chrysin may be a potential compound for adjuvant treatment of human SCC.

## Material and Methods

### Materials

Antibodies used in the present experiments were listed in Table 1. Chrysin, Lipofectamine 2000, luteolin, LY-294002, and human recombinant Akt were obtained from Tokyo Kasei Kogyo (Tokyo, Japan), Thermo Fisher Scientific (Rockford, IL, USA), INDOFINE Chemical Company (Hillsborough, NJ, USA), BIOMOL Research Laboratories (Plymouth Meeting, PA, USA), and ProSpec-Tany TechnoGene (Rehovot, Israel), respectively. CDDP, DXR, and ScreenFect A were from Wako Pure Chemical (Osaka, Japan). All other reagents were of the highest purity commercially available.

**Table 1:** Lists of antibodies.

Name	Sources	Catalog No.	Suppliers
p-Akt	Rabbit	4060	Cell Signaling Technology (Beverly, MA, USA)
Akt	Rabbit	4691	
ERK1/2	Rabbit	4695	
ABCC2	Rabbit	4446	
p-PDK1	Rabbit	S241	
PDK1	Rabbit	3062	
β-Actin	Goat	sc-1615	Santa Cruz Biotechnology (Santa Cruz, CA, USA)
p-ERK1/2	Rabbit	sc-16982R	
ABCB1	Rabbit	GTX108354	GeneTex (Irvine, CA, USA)
ABCC1	Rabbit	GTX116046	
ABCG2	Rabbit	GTX100437	
ZO-1	Mouse	33–9100	Thermo Fisher Scientific (Rockford, IL, USA)

### 2D and 3D Cell culture

RERF-LC-AI cells (RIKEN BRC through the National Bio-Resource Project of the MEXT, Ibaraki, Japan) were cultured as described previously^10^. For 3D culture, the cells were plated at densities of 1 × 10^4^ cells/well on PrimeSurface96V multi-well plates (Sumitomo Bakelite, Tokyo, Japan). After culturing for 96 h, the size and viability of spheroids were measured as described previously^10^. The fluorescence intensities of DXR and LOX-1, a hypoxia probe, were calculated using ImageJ software.

### siRNA and transfection

siRNAs for negative control and CLDNs were obtained from Santa Cruz and Sigma-Aldrich, respectively. The siRNAs were transfected into 2D and 3D cultured cells using Lipofectamine 2000 and ScreenFect A, respectively.

### Cell viability assay in 2D culture

Cells were seeded at densities of 7 × 10^3^ cells on 96-well flat bottomed plates. Chrysin, luteolin, and anticancer drugs were applied for 24 h in FCS-free media. The cell viability was measured using a Premix WST-1 Cell Proliferation Assay Kit (Takara, Otsu, Japan).

### Isolation of total RNA and quantitative real-time polymerase chain reaction

Total RNA was extracted from cells using TRI reagent (Sigma-Aldrich). To compare the expression of CLDNs between human normal lung and RERF-LC-AI cells, we used three independent mRNAs of normal lung tissue (Clontech Laboratories, Mountain View, CA, USA, Agilent Technologies, Santa Clara, CA, USA, and BioChain Institute, Hayward, CA, USA). The expression of CLDNs between human normal lung and SCC tissues were investigated using Lung Cancer cDNA Array II and V (OriGene, Rockville, MD, USA). Reverse transcription was carried out using a ReverTra Ace qPCR RT Kit (Toyobo Life Science, Osaka, Japan). Quantitative real-time PCR was performed using an Eco Real-Time polymerase chain reaction (PCR) system (AS One, Osaka, Japan) with a THUNDERBIRD SYBR qPCR Mix (Toyobo Life Science). The primers used for PCR are listed in Table 2. The relative change in mRNA expression was calculated as described previously^10^.

**Table 2:** Primers for real-time PCR.

Genes	Direction	Sequence
*CLDN1*	Sense	5′-ATGAGGATGGCTGTCATTGG-3′
*CLDN1*	Antisense	5′-ATTGACTGGGGTCATAGGGT-3′
*CLDN11*	Sense	5′-ACGGGGCTGTACCACTGCAA-3′
*CLDN11*	Antisense	5′-CAGGACCGAGGCAGCAATCATCAG-3′
β*-Actin*	Sense	5′-CCTGAGGCACTCTTCCAGCCTT-3′
β*-Actin*	Antisense	5′-TGCGGATGTCCACGTCACACTTC-3′

### Sodium dodecyl sulfate-polyacrylamide gel electrophoresis and western blotting

Confluent cells were scraped into cold phosphate-buffered saline and precipitated by centrifugation. Then, the cells were lysed in a RIPA buffer (150 mM NaCl, 50 mM Tris-HCl (pH 8.0), 1% Triton X-100, 0.1% sodium dodecyl sulfate (SDS), 0.5 mM EDTA) supplemented with a protease inhibitor cocktail (Sigma-Aldrich), and sonicated for 20 s. Nuclear fraction was removed by centrifugation at 6,000 × g for 5 min. The resultant supernatants were used as cell lysates. SDS-polyacrylamide gel electrophoresis and western blotting were carried out as described previously^10^.

### Immunoprecipitation

Cell lysates were incubated with Protein G-sepharose (GE Healthcare, Bucks, UK) and anti-PDK1 antibodies for 16 h at 4 °C using a rotator. In the case using biotinylated-chrysin, cell lysates were incubated with or without biotinylated-chrysin in the presence of avidin agarose (Thermo Fisher Scientific). Immunoprecipitants were washed four times with an immunoprecipitation buffer (150 mM NaCl, 0.5 mM EDTA, 0.5% Triton X-100, 50 mM Tris-HCl (pH 7.4), and a protease inhibitor cocktail)and then subjected to SDS-polyacrylamide gel electrophoresis.

### Confocal microscopy

Immunofluorescence measurements were carried out as described previously^10^.

### Measurement of transepithelial permeability

Cells were seeded at densities of 5 × 10^4^ cells on Transwells (0.4 μm pore size, 12 mm diameter) with polyester membrane inserts (Corning Incorporated, Corning, NY, USA). TER and paracellular flux of lucifer yellow and DXR were measured as describe previously^10^.

### Synthesis of biotinylated chrysin

#### Preparation of chrysin azide

To a solution of 7-*O*-carboxymethyl chrysin^43^ (100 mg, 0.32 mmol), 3-azidopropan-1-amine^44^ (65 mg, 0.61 μmol), and EDCI (117 mg, 0.61 mmol) in DMF (2.0 mL) was added and DMAP (6.0 mg, 49 μmol) at room temperature (rt) in an Ar atmosphere. After being stirred for 12 h at rt, DMAP (60 mg, 0.49 mmol) was further added, and the reaction mixture was warmed to 70 °C. Even after stirring for 6 h at 70 °C, 7-*O*-carboxymethyl chrysin was still present. Therefore, 3-azidopropan-1-amine (40 mg, 0.40 μmol) was added and stirred for 15 h at 70 °C. 3-Azidopropan-1-amine (40 mg, 0.40 μmol) was further added and stirred for 6 h at 80 °C. The reaction mixture was diluted with AcOEt, washed with saturated aqueous NH_4_Cl and brine. The organic layer was dried over Na_2_SO_4_, filtered, and evaporated *in vacuo* to give a residue. The residue was crystallized from hexane-CHCl_3_ to afford chrysin azide as colorless prisms (55 mg, 29%).

Colorless prisms (hexane-CHCl_3_); Mp 144–145 °C. ^1^H nuclear magnetic resonance (NMR; 300 MHz, CDCl_3_) δ (quin, *J* = 6.5 Hz, 2H), 3.41 (t, *J* = 6.5 Hz, 2H), 3.48 (q, *J* = 6.5 Hz, 2H), 4.58 (s, 2H), 6.41 (d, *J* = 2.3 Hz, 1H), 6.53 (d, *J* = 2.3 Hz, 1H), 6.70 (s, 1H), 6.65–6.80 (m, 2H), 7.50–7.61 (m, 3H), 7.83–7.93 (m, 2H), 12.77 (s, 1H); ^13^C NMR (75 MHz, CDCl_3_) δ 28.7, 37.0, 49.4, 67.4, 93.0, 99.0, 106.1, 106.7, 126.4, 129.2, 131.1, 132.1, 157.8, 162.5, 162.7, 164.4, 167.2, 182.5; IR (ATR) 2085, 1658, 1606 cm^-1^; MS (FAB) *m/z* 395 (M + H)^+^; HRMS (FAB) *m/z* calcd for C_20_H_19_N_4_O_5_ (M + H)^+^ 395.1355, found 395.1356.

#### Preparation of biotinylated chrysin

To a solution of chrysin azide (45 mg, 0.11 mmol), biotin-alkyne conjugate^45^ (32 mg, 0.11 μmol), and CuTC (4.3 mg, 23 μmol) in CH_2_Cl_2_ (3.0 mL)-MeOH (0.3 mL) was added and stirred for 12 h at rt in an Ar atmosphere. The reaction mixture was evaporated in vacuo to give a residue. The residue was successively washed with H_2_O, CHCl_3_, and MeOH. The washed residue was dissolved in DMF (2.0 mL), and silica gel for column chromatography (2.0 g) was added and evaporated in vacuo to afford the residue adsorbed on silica gel. This residue was applied to silica gel column and eluted with CHCl_3_/MeOH (5:1) to give a solid. This solid was further washed with CHCl_3_ to give biotinylated chrysin as pale yellow solid (20 mg, 26%).

^1^H NMR (300 MHz, DMSO-*d*_6_) δ 1.21–1.35 (m, 2H), 1.38–1.67 (m, 4H), 1.90 (quin *J* = 6.9 Hz, 2H), 2.09 (t, *J* = 7.4 Hz, 2H), 2.57 (d, *J* = 12.5 Hz, 1H), 2.81 (dd, *J* = 5.0 Hz, 12.5 Hz, 1H), 3.04–3.12 (m, 1H), 3.15–3.23 (m, 2 H), 4.08–4.15 (m, 1H), 4.22–4.40 (m, 5H), 4.66 (s, 2H), 6.36 (s, 1H), 6.41 (s, 1H), 6.46 (d, *J* = 2.2 Hz, 1H), 6.85 (d, *J* = 2.2 Hz, 1H), 7.07 (s, 1H), 7.55–7.68 (m, 3H), 7.91 (s, 1H), 8.07–8.13 (m, 2H), 8.21–8.33 (m, 2H), 12.82 (s, 1 H); ^13^C NMR (125 MHz, DMSO-*d*_6_) δ 25.2, 28.0, 28.2, 29.9, 34.1, 35.0, 35.8, 47.1, 55.4, 59.2, 61.0, 67.2, 79.0, 79.2, 93.7, 98.9, 105.4, 105.5, 122.9, 126.6, 129.3, 130.7, 132.3, 145.1, 157.4, 162.8, 163.7, 163.8, 167.1, 172.1, 182.3; IR (ATR) 1702, 1658, 1614 cm^-1^; MS (ESI) *m/z* 676 (M + H)^+^; HRMS (ESI) *m/z* calcd for C_33_H_38_N_7_O_7_S (M + H)^+^ 676.2548, found 676.2542.

### A quartz-crystal microbalance analysis

Avidin was deposited on the Au electrode of a QCM twin sensor chip ((Nihon Dempa Kogyo, Tokyo, Japan), which consists of two channels (CH1 and CH2). CH1 and CH2 were modified with biotin and biotinylated-chrysin, respectively. Human recombinant Akt (5 μg/ml) was injected into the flow cell at a flow rate of 50 μL/min. Detection of the association between chrysin and Akt was carried using a QCM sensor system. Sensor response was measured by subtracting the frequency shifts of CH2 from CH1.

### Statistical analysis

Results are presented as means ± standard error mean. Statistical analyses were performed using KaleidaGraph version 4.5.1 software (Synergy Software, PA, USA) as described previously^10^. Significant differences were accepted at *p* < 0.05.

## Supplementary information

Highlights

Dataset 1
