# Supplementary material for: Chrysin enhances anticancer drug-induced toxicity mediated by the reduction of claudin-1 and 11 expression in a spheroid culture model of lung squamous cell carcinoma cells
Source: Sci Rep. 2019 Sep 24;9:13753. doi: 10.1038/s41598-019-50276-z (PMC6760125; doi:10.1038/s41598-019-50276-z)
Supplement: Supplementary file 1 — Highlights [file 41598_2019_50276_MOESM1_ESM.doc]

Highlights

- Claudin-1 and -11 are highly expressed in human squamous cell carcinoma cells.
- Chrysin decreased the expression levels of claudin-1 and -11 mediated by the inhibition of Akt.
- Immunoprecipitation and quartz-crystal microbalance assays showed that chrysin binds directly to Akt.
- Chrysin enhanced anticancer drug-induced toxicity in a three dimensional spheroid model.
- Chrysin may be a potential compound for adjuvant treatment of human squamous cell carcinoma.
