# Supplementary material for: Chrysin enhances anticancer drug-induced toxicity mediated by the reduction of claudin-1 and 11 expression in a spheroid culture model of lung squamous cell carcinoma cells
Source: Sci Rep. 2019 Sep 24;9:13753. doi: 10.1038/s41598-019-50276-z (PMC6760125; doi:10.1038/s41598-019-50276-z)
Supplement: Supplementary file 2 — Dataset 1 [file 41598_2019_50276_MOESM2_ESM.pdf]

Supplementary Information

Chrysin enhances anticancer drug-induced toxicity mediated by the reduction of claudin-1 and 11 expression in a spheroid culture model of lung squamous cell carcinoma cells

Ryohei Maruhashi[1#], Hiroaki Eguchi[1#], Risa Akizuki[1], Shohei Hamada[2], Takumi Furuta[2], Toshiyuki Matsunaga[1], Satoshi Endo[1], Kenji Ichihara[3], and Akira Ikari[1*]

[1] From the Laboratory of Biochemistry, Department of Biopharmaceutical Sciences, Gifu Pharmaceutical University, Gifu 501-1196, Japan
[2] Department of Pharmaceutical Chemistry, Kyoto Pharmaceutical University, Yamashina-ku, Kyoto 607-8414, Japan
[3] Nagaragawa Research Center, API Co., Ltd., Gifu 502-0071, Japan

Running title: Chrysin enhances anticancer activity in spheroid cells

[#] These two authors have contributed equally to this study

To whom correspondence should be addressed: Akira Ikari, Ph.D.
Laboratory of Biochemistry, Department of Biopharmaceutical Sciences,
Gifu Pharmaceutical University, 1-25-4 Daigaku-nishi, Gifu 501-1196, Japan,
Tel: +81-58-230-8124; Fax: +81-58-230-8124; E-mail: ikari@gifu-pu.ac.jp

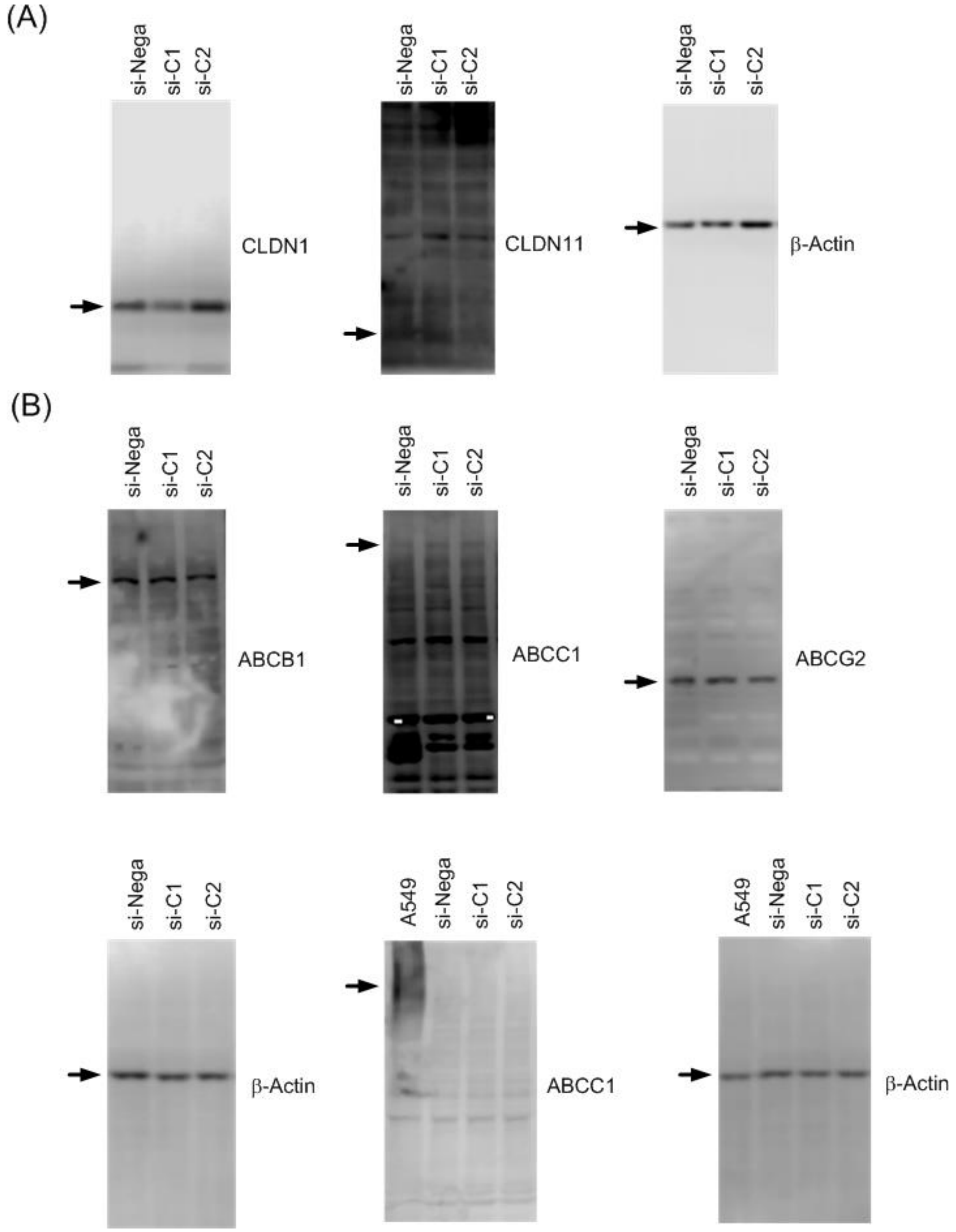

Supplementary Figure S1. Full images of the blots from figure 2.

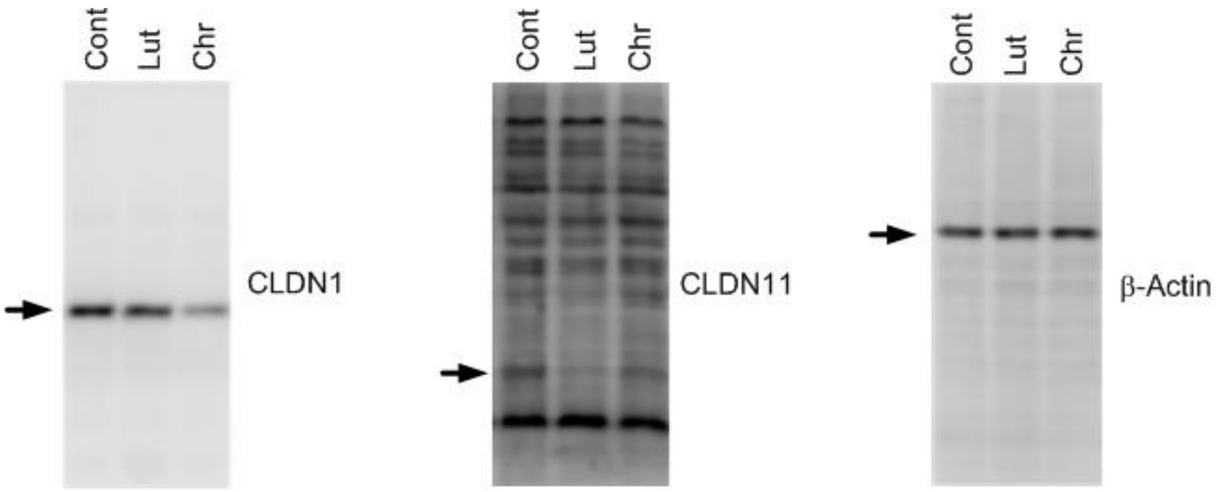

Supplementary Figure S2. Full images of the blots from figure 5B.

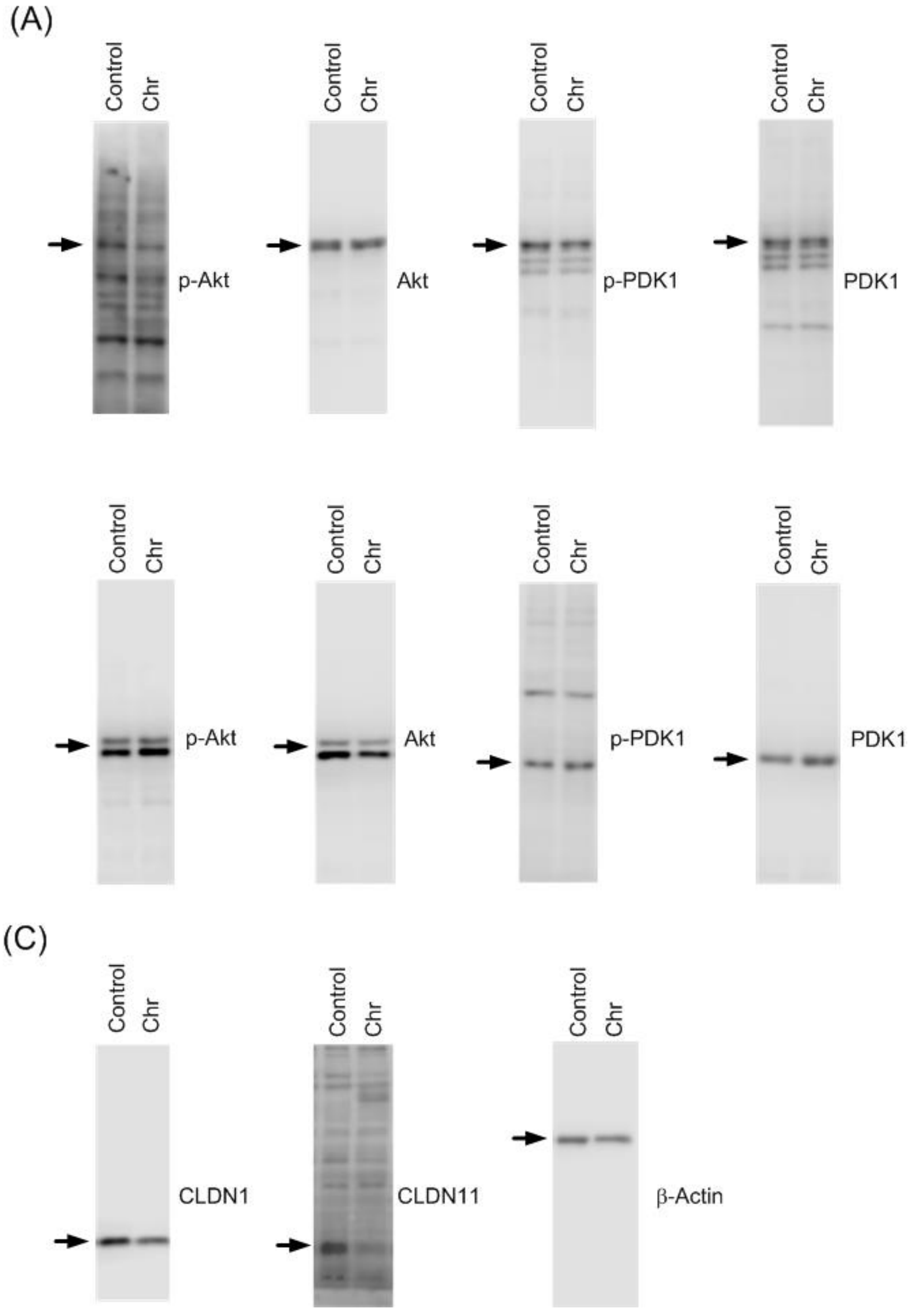

Supplementary Figure S3. Full images of the blots from figure 6.

(B)

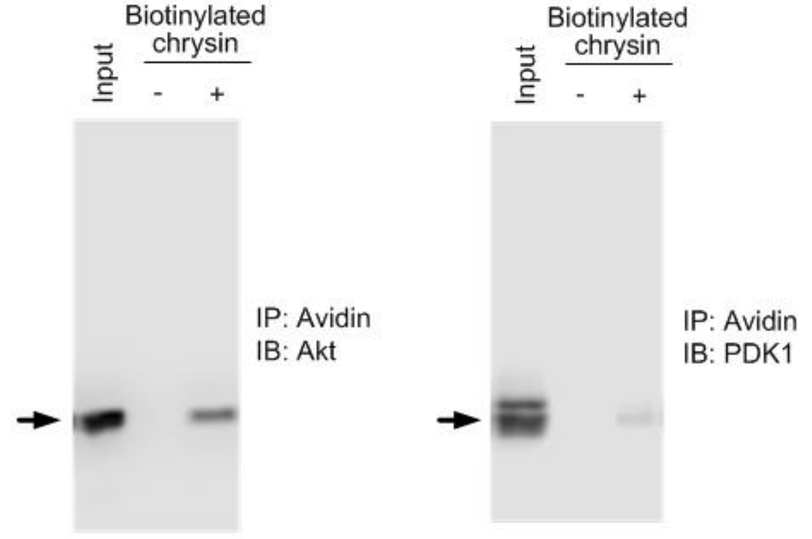

(D)

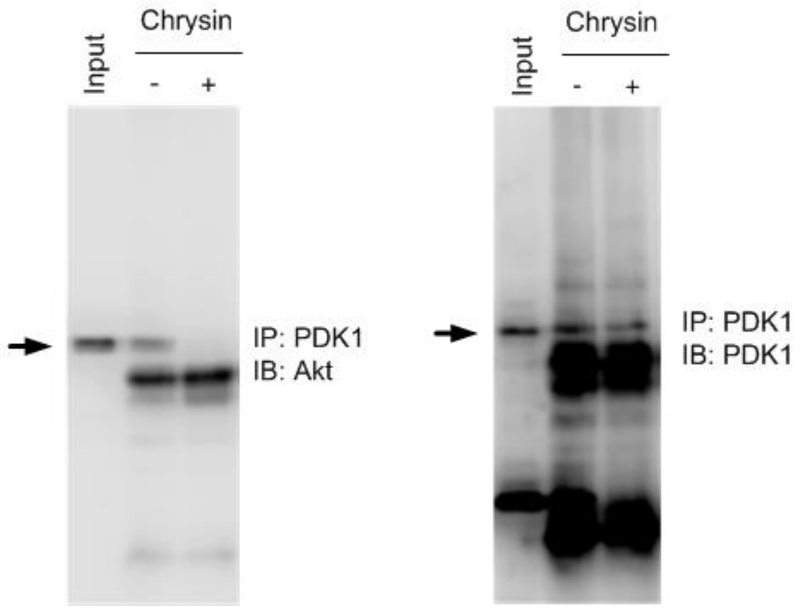

Supplementary Figure S4. Full images of the blots from figure 7.
